# Peripheral Hybrid CB_1_R and iNOS Antagonist MRI-1867 Displays Anti-Fibrotic Efficacy in Bleomycin-Induced Skin Fibrosis

**DOI:** 10.3389/fendo.2021.744857

**Published:** 2021-09-28

**Authors:** Charles N. Zawatsky, Joshua K. Park, Jasmina Abdalla, George Kunos, Malliga R. Iyer, Resat Cinar

**Affiliations:** ^1^ Section on Fibrotic Disorders, National Institute on Alcohol Abuse and Alcoholism, National Institutes of Health, Rockville, MD, United States; ^2^ Laboratory of Physiologic Studies, National Institute on Alcohol Abuse and Alcoholism, National Institutes of Health, Rockville, MD, United States; ^3^ Section on Medicinal Chemistry, National Institute on Alcohol Abuse and Alcoholism, National Institutes of Health, Rockville, MD, United States

**Keywords:** endocannabinoids, skin fibrosis, bleomycin, ATP-binding cassette transporters, P-gp (P-glycoprotein), peripheral CB1R antagonist, cannabinoid (CB) receptor 1, polypharmacology

## Abstract

Scleroderma, or systemic sclerosis, is a multi-organ connective tissue disease resulting in fibrosis of the skin, heart, and lungs with no effective treatment. Endocannabinoids acting *via* cannabinoid-1 receptors (CB_1_R) and increased activity of inducible NO synthase (iNOS) promote tissue fibrosis including skin fibrosis, and joint targeting of these pathways may improve therapeutic efficacy. Recently, we showed that in mouse models of liver, lung and kidney fibrosis, treatment with a peripherally restricted hybrid CB_1_R/iNOS inhibitor (MRI-1867) yields greater anti-fibrotic efficacy than inhibiting either target alone. Here, we evaluated the therapeutic efficacy of MRI-1867 in bleomycin-induced skin fibrosis. Skin fibrosis was induced in C57BL/6J (B6) and Mdr1_a/b_-Bcrp triple knock-out (KO) mice by daily subcutaneous injections of bleomycin (2 IU/100 µL) for 28 days. Starting on day 15, mice were treated for 2 weeks with daily oral gavage of vehicle or MRI-1867. Skin levels of MRI-1867 and endocannabinoids were measured by mass spectrometry to assess target exposure and engagement by MRI-1867. Fibrosis was characterized histologically by dermal thickening and biochemically by hydroxyproline content. We also evaluated the potential increase of drug-efflux associated ABC transporters by bleomycin in skin fibrosis, which could affect target exposure to test compounds, as reported in bleomycin-induced lung fibrosis. Bleomycin-induced skin fibrosis was comparable in B6 and Mdr1_a/b_-Bcrp KO mice. However, the skin level of MRI-1867, an MDR1 substrate, was dramatically lower in B6 mice (0.023 µM) than in Mdr1_a/b_-Bcrp KO mice (8.8 µM) due to a bleomycin-induced increase in efflux activity of MDR1 in fibrotic skin. Furthermore, the endocannabinoids anandamide and 2-arachidonylglycerol were elevated 2-4-fold in the fibrotic *vs*. control skin in both mouse strains. MRI-1867 treatment attenuated bleomycin-induced established skin fibrosis and the associated increase in endocannabinoids in Mdr1_a/b_-Bcrp KO mice but not in B6 mice. We conclude that combined inhibition of CB_1_R and iNOS is an effective anti-fibrotic strategy for scleroderma. As bleomycin induces an artifact in testing antifibrotic drug candidates that are substrates of drug-efflux transporters, using Mdr1_a/b_-Bcrp KO mice for preclinical testing of such compounds avoids this pitfall.

## Introduction

Scleroderma, or systemic sclerosis (SSc), is a connective tissue disease with multiple clinical manifestations, including autoimmunity, vascular dysfunction, and tissue fibrosis (1), and a prevalence in the United States of around 240 cases per 1 million adults (2). Scleroderma is a complex, heterogeneous disease with clinical forms ranging from limited skin involvement (limited cutaneous systemic sclerosis) to forms with diffuse skin sclerosis and severe and often progressive internal organ involvement (diffuse cutaneous systemic sclerosis) (3). Pulmonary fibrosis and interstitial lung diseases (ILD) occur in about 60% of patients, contributing to mortality (4), while dermal fibrosis causes significant morbidity in scleroderma (5, 6). In the absence of approved therapies, there is an unmet need for identifying new targets and treatment strategies. Due to the complex and multifactorial pathogenesis of scleroderma, targeting multiple signaling pathways may be essential for effective treatment (7).

Inducible nitric oxide synthase (iNOS) is an enzyme encoded by the Nos2 gene and is responsible for generating pro-inflammatory reactive nitrogen species (8). The relevance of iNOS as a target is based on evidence for overproduction of NO in the pathogenesis of SSc (9, 10). In SSc, the expression of iNOS in the endothelium, smooth muscle cells, fibroblasts, macrophages and many other cell types is robustly induced by inflammatory mediators and cytokines and its activity is increased at inflammatory sites (8). The iNOS-mediated formation of NO is increased in inflammatory cells such as macrophages or activated fibroblasts (10). Immunohistological studies of scleroderma skin show that disease progression involves iNOS upregulation (11). Previous studies also demonstrate that SSc lung macrophages express high levels of iNOS and produce a high quantity of ONOO^-^ anions (11). In SSc patients, increased production of NO is suggested by the increased expression of iNOS in endothelial cells, fibroblasts and mononuclear cells infiltrating the fibrotic skin (12) as well as in alveolar macrophages (13). The role of NO synthases and especially iNOS is elegantly dissected by the work of Cotton et al., which proposes an active role of iNOS-induced NO production in endothelial cell damage and advances the concept of iNOS inhibition as a viable therapeutic strategy for SSc (14).

An additional target that is becoming increasingly relevant in the modulation of fibrotic responses is the endocannabinoid system. Endocannabinoids are lipid-signaling molecules that act through cannabinoid receptors CB_1_ and CB_2_. Endocannabinoids acting *via* CB_1_R promote fibrosis in multiple organs including skin (15), liver (16–18), kidney (19), and heart (20). Besides, CB_1_R activation is pro-inflammatory (21). Increased CB_1_R activity has been linked to different forms of pulmonary fibrosis such as radiation-induced pulmonary fibrosis (22), idiopathic pulmonary fibrosis (23) and Hermansky-Pudlak syndrome pulmonary fibrosis (HPSPF) (24). Conversely, CB_1_R antagonism prevents fibroblast activation and exerts potent antifibrotic effects in skin fibrosis (25). The role of CB_1_R as a pro-fibrotic receptor has also been confirmed in fatty acid amide hydrolase knock-out mice, in which elevated levels of anandamide induced skin fibrosis in a CB_1_R-dependent manner (26).

Bleomycin is widely used to induce fibrosis in rodent models of fibrotic disorders. We have earlier reported that bleomycin induces drug-efflux associated ABC transporters in the lung, which limits exposure of the fibrotic tissue to drug candidates that are substrates of such transporters. Here we show that this pitfall could be avoided by establishing bleomycin-induced skin fibrosis in $\text{Mdr}1_{\text{a}/\text{b}}^{\left(-/-\right)}$ -Bcrp^(-/-)^ triple knock-out mice and using this model to reveal the antifibrotic therapeutic efficacy of the peripherally restricted hybrid CB_1_R/iNOS antagonist MRI-1867, a known substrate of drug efflux transporters.

## Materials and Methods

### Chemicals

*S*-MRI-1867, referred to as MRI-1867, was synthesized as described previously (16). Rimonabant was obtained from the National Institute of Drug Abuse Drug Supply Program (Research Triangle Park, NC, USA). Pharmaceutical grade bleomycin was obtained from Hospira. All other chemicals were from Sigma-Aldrich.

### Animals

All animal procedures were conducted in accordance with the rules and regulations of the Institutional Animal Care and Use Committee of the National Institutes of Alcohol Abuse and Alcoholism (NIAAA). C57BL/6J mice were purchased from the Jackson Laboratory (Bar Harbor, ME, USA). $\text{Mdr}1_{\text{a}/\text{b}}^{\left(-/-\right)}$ -Bcrp^(-/-)^ mice were purchased from Taconic (Rensselaer, NY, USA). Animals were housed individually under a 12-hour light/dark cycle and fed a standard diet, *ad libitum* (Teklad NIH-31; Envigo, Huntingdon, UK).

### Bleomycin-Induced Skin Fibrosis

**Figure 1:**
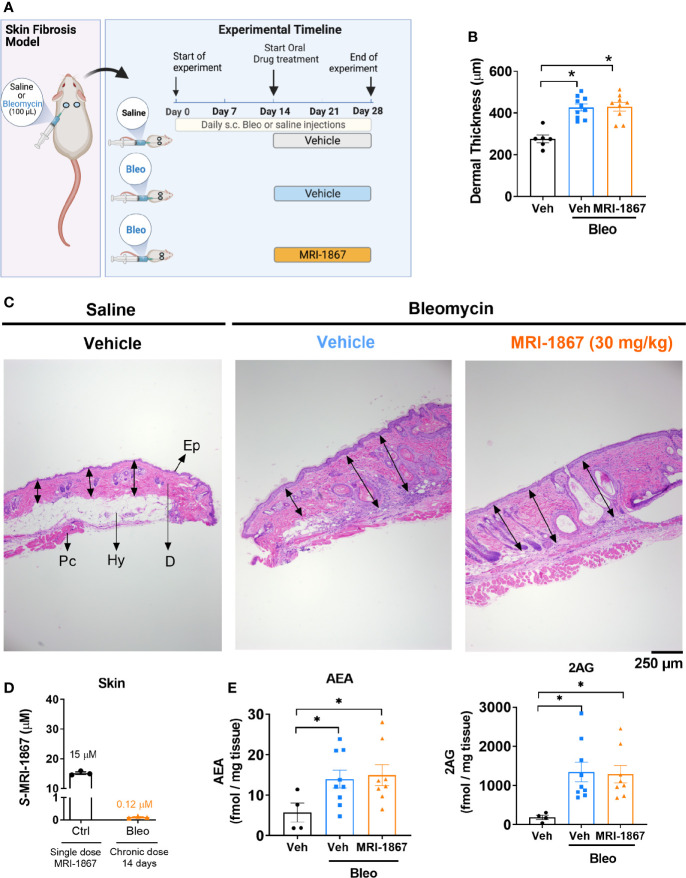
Bleomycin-induced skin fibrosis minimizes skin exposure and target engagement by MRI-1867 in C57BL6/J mice. **(A)** Schematic presentation of bleomycin-induced skin fibrosis development and therapeutic treatment regime. **(B)** Dermal thickness quantification for histological images. **(C)** Representative histological images from skin biopsies stained with H&E Ep, Epidermis; D, Dermis; Hy, Hypodermis and adipose tissue; Pc, Panninculus carnosus. Double arrow indicates dermal length that used for quantification. **(D)** Levels of MRI-1867 in control and bleomycin-treated skin biopsy specimens following acute or 14-day chronic administration of MRI-1867 at 10 mg/kg, respectively. **(E)** Levels of endocannabinoids AEA and 2AG in skin biopsies. Data represent means ± SEM from 3-10 mice per group. Data were analyzed by 1-way ANOVA followed by Dunnett’s multiple comparisons test. Significant difference from corresponding values in controls (saline and vehicle treated group) (*P < 0.05).

This study used male C57BL/6J and $\text{Mdr}1_{\text{a}/\text{b}}^{\left(-/-\right)}$ -Bcrp^(-/-)^ (KO) mice, ranging from 16-20 weeks of age, with an average initial body weight of 28.5 g. Mice received daily 100µL subcutaneous injections dorsal to each scapula for 28 days of either vehicle (sterile 0.9% saline), or 2 IU of bleomycin (bleo) to induce skin fibrosis (**Figure 1A**).

### Therapeutic Intervention

10 mg/kg MRI-1867 or its drug-free vehicle (5% DMSO, 5% Tween 80 in 0.9% saline) were administered by oral gavage either as a single dose in control mice or starting on day 15 and ending on day 28 of bleomycin treatment in bleomycin-treated mice. Mice were sacrificed 1 h following the single dose (control) or the last daily dose of MRI-1867 (bleo-treated mice).

### Dermal Thickness Histological Assessment

Skin tissues were fixed in 10% neutralized formalin solution for 24 hours, embedded in paraffin, and sectioned (4μm) onto glass slides. Sections were counterstained with hematoxylin and eosin and imaged with an Olympus BX41 microscope. Images were taken at 4x magnification to cover 6 mm diameter skin biopsy section from each skin section. Skin thickness was quantified by measuring the length of a straight line between the limits of dermal tissue using ImageJ software. Data points reflect the means of 7-13 independent measurements from the same mouse.

### Hydroxyproline Measurements by LC-MS/MS

Skin fibrosis was quantified by measuring hydroxyproline (Hyp) content of skin biopsies consisting of epidermis and dermis, using LC-MS/MS as described previously (23) with slight modifications. Briefly, 15-30 mg skin tissue was homogenized in 600 μL of ice-cold 0.1 N perchloric acid (PCA) then 200 µl of homogenate was aliquoted and prepared for endocannabinoid and MRI-1867 measurements as detailed below. One mL 12 N HCl was added to the remaining 400 µL skin homogenate and the homogenate was hydrolyzed at 100°C for 4 hours. Hydrolyzed samples were vortexed and centrifuged at 10,000 g for 10 minutes, and 5 μL hydrolysate was diluted 200-fold by the addition of 990 μL of 0.1 N PCA and 5 μL of L-Proline-^13^C_5_,^15^N as internal standard. Liquid chromatography tandem mass spectrometry (LC-MS/MS) analyses were conducted on an Agilent 6410 triple quadrupole mass spectrometer (Agilent Technologies) coupled to an Agilent 1200 LC system. 4-Hydroxyproline was separated using an Intrada Amino Acid column, 50 × 3 mm, 3 μm (Imtakt) at 40°C. Mobile phases consisted of acetonitrile/tetrahydrofuran/25 mM ammonium formate/formic acid = 9:75:16:0.3 (v/v/v/v) (phase A) and acetonitrile/100 mM ammonium formate = 20:80 (v/v) (phase B). Gradient elution (600 μL/min) was initiated and held at 0% B for 3 minutes, followed by a linear increase to 17% B by 6.5 minutes. This was followed by a step increase to 100% B, which was held until 10 minutes after the gradient had begun, and then by a linear decrease to 0% B by 11 minutes, which was held until 13 minutes after the gradient had begun. The mass spectrometer was set for electrospray ionization operated in positive ion mode. The source parameters were as follows: capillary voltage, 4,000 V; gas temperature, 330°C; and drying gas, 8 L/min. Nitrogen was used as the nebulizing gas. Collision-induced dissociation (CID) was conducted using nitrogen. Hydroxyproline level was analyzed by multiple reaction monitoring. L-Proline-^13^C_5_,^15^N (Sigma, cat#608114) was used as the internal standard. The molecular ion and fragments for hydroxyproline were measured as follows: m/z 132.1→86 and 132.1→68 (CID energy: 8 V and 20 V, respectively). Skin levels of hydroxyproline were determined against a standard curve, using trans-4-hydroxy-L-proline as standard (Sigma-Aldrich). Values are expressed as nmol/mg wet tissue.

### Endocannabinoid Extraction and Analysis

Skin homogenate (200 µL) described in the hydroxyproline measurement section was used and transferred in 0.5 mL of ice-cold methanol/Tris buffer (50 mM, pH 8.0), 1:1, containing 7 ng of [^2^H_4_] arachidonoyl ethanolamide ([^2^H_4_] AEA) as internal standard. Homogenates were extracted three times with CHCl_3_: MeOH (2:1, vol/vol), dried under nitrogen flow, and reconstituted with MeOH after precipitating proteins with ice-cold acetone. LC-MS/MS analyses were conducted on an Agilent 6410 triple quadrupole mass spectrometer (Agilent Technologies) coupled to an Agilent 1200 LC system. Analytes were separated using a Zorbax SB-C18 rapid-resolution HT column. Gradient elution mobile phases consisted of 0.1% formic acid in H_2_O (phase A) and 0.1% formic acid in MeOH (phase B). Gradient elution (250 μL/min) was initiated and held at 10% B for 0.5 min, followed by a linear increase to 85% B at 1 min and maintained until 12.5 min, then increased linearly to 100% B at 13 min and maintained until 14.5 min. The mass spectrometer was set for electrospray ionization operated in positive ion mode. The source parameters were as follows: capillary voltage, 4,000 V; gas temperature, 350°C; drying gas, 10 L/min; nitrogen was used as the nebulizing gas. Collision-induced dissociation was performed using nitrogen. Levels of each compound were analyzed by multiple reaction monitoring. The molecular ion and fragment for each compound were measured as follows: m/z 348.3→62.1 for AEA, m/z 352.3→66.1 for [^2^H_4_] AEA, and m/z 379.3→91.1 for 2-arachidonoylglycerol (2-AG). Analytes were quantified using MassHunter Workstation LC/QQQ Acquisition and MassHunter Workstation Quantitative Analysis software (Agilent Technologies). Levels of AEA and 2-AG in the samples were measured against standard curves. Values are expressed as fmol/mg wet tissue.

### Tissue Levels of MRI-1867

Two hundred μL of skin homogenate described above and 30 µl serum were used and extracted as described above for endocannabinoid extraction. MRI-1867 levels were determined by LC-MS/MS using an Agilent 6410 triple quadrupole mass spectrometer (Agilent Technologies) coupled to an Agilent 1200 LC system (Agilent Technologies). Levels of MRI-1867 were analyzed by multiple reaction monitoring. The molecular ion and fragments were measured as follows: m/z 548.1→145 and 548.1→257.1 for MRI-1867 (CID energy: 56 V and 24 V, respectively). The amounts of MRI-1867 in the samples were determined against a standard curve. Values are expressed as µM.

### Immunohistochemistry

Immunohistochemistry was performed as previously described (27). Tissue sections were incubated overnight at 4°C in an optimized blocking solution: 5% blotting-grade milk (Bio-Rad, Hercules, CA) and 5% horse serum (Vector Laboratories, Burlingame, CA, USA) diluted in double distilled H_2_O. Rabbit monoclonal anti-P-Glycoprotein antibody (Abcam, Cat. ab170904) was diluted (1:1000 for all mouse tissues) in 5% milk and 5% horse serum as described above for overnight incubation at 4°C. The appropriate secondary antibody, anti-rabbit IgG made in horse (Cat. MP-7401, Vector Laboratories) was not diluted, and incubated at room temperature for 1 hour. Positive immunoreactivity was revealed *via* chromogenic detection with ImmPACT DAB Peroxidase (HRP) Substrate (SK-4105, Vector Laboratories) with incubating 3 minutes, then counterstained with hematoxylin (Gills Formula, Vector Laboratories) for five seconds and covered with a coverslip. Images were captured with a BX41 bright-field microscope (Olympus, Center Valley, PA, USA). Immunostaining intensity was quantified by using ImageJ software (NIH Public Domain) by a person blind to sample ID. Images were taken at 20x magnification from at least 5 randomly selected areas per skin specimen.

### Statistical Analysis

Statistical analysis was performed using GraphPad Prism 8 (GraphPad Software Inc.). Normality test was performed by Prism 8 to determine whether samples show normal distribution. Then, one-way ANOVA followed by Dunnett’s multiple comparisons test was performed. *P* < 0.05 was considered significant. In multiple comparison post-hoc test, control groups were designated to address each statistical question as indicated in figure legends for statistical significance.

## Results

### Bleomycin Significantly Attenuates Skin Exposure of MRI-1867 in C57BL/6J Mice due to Increased Expression of P-Glycoprotein

One of the hallmarks of systemic sclerosis is skin thickening due to fibrosis. Bleomycin is commonly used as an exogenous inducer of fibrosis in murine models of skin and pulmonary fibrosis. In this study, skin fibrosis was induced by daily subcutaneous injection of bleomycin for 28 days as detailed in the methods and as depicted in **Figure 1A**. We recently demonstrated that bleomycin induces drug efflux transporters in murine lungs and that this mechanism compromises lung exposure to chemical compounds that happen to be substrates (27). To determine whether a similar mechanism is triggered by bleomycin in the skin, we first assessed the skin levels of the peripherally restricted hybrid CB_1_R/iNOS inhibitor MRI-1867, a substrate of P-gp (16), in a bleomycin-induced murine model of skin fibrosis.

**Figure 2:**
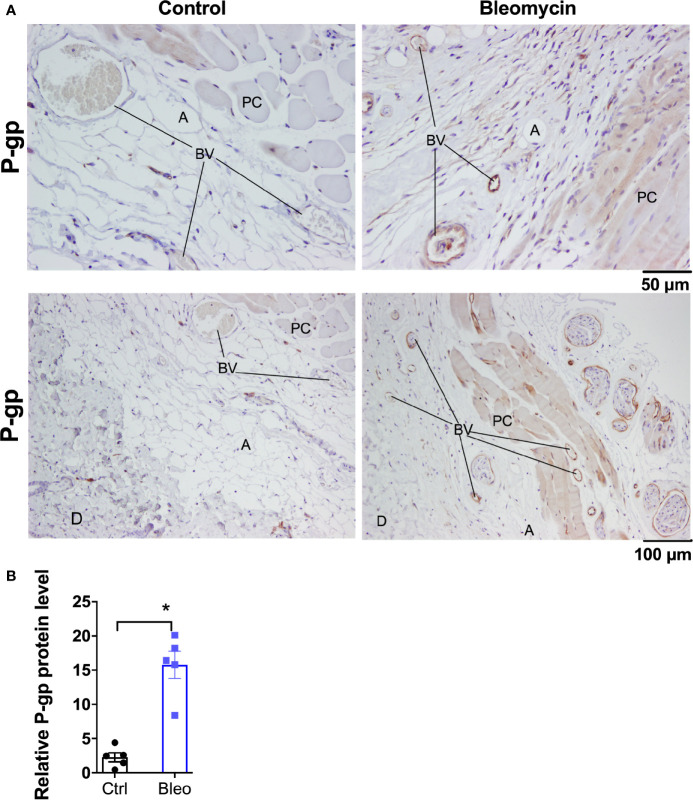
Bleomycin induces drug efflux transporter P-gp in skin of C57BL6/J mice. **(A)** Representative histological images for P-gp immunohistochemistry staining in skin biopsies of control and bleomycin-induced fibrotic skin. A, adipocytes; BV, blood vessels; D, Dermis; PC, Panninculus carnosus. **(B)** Quantification of P-gp protein expression in skin biopsies. Data represent mean ± SEM from five subjects in each group. Data were analyzed by t-test for comparison of histological scoring. * (p <0.05) indicates significant difference from the control group.

Ten mg/kg is the maximally effective dose of MRI-1867 for peripheral CB_1_R antagonism (16), and this dose was previously shown to achieve dual-target inhibition of CB_1_R and iNOS in lung and kidney fibrosis (23, 28). MRI-1867 was administered by oral gavage either as a single dose in control mice or daily for 14 days in bleomycin-treated mice (**Figure 1A**). Bleomycin (2U/day for 28 days) induced skin fibrosis in C57BL/6J mice, as quantified by measuring dermal thickness (**Figures 1B, C**). Notably, the levels of MRI-1867 in fibrotic skin tissue were significantly lower (0.12 µM) than in healthy control skin (15 µM after single dosing) (**Figure 1D**). Skin levels of anandamide (AEA) and 2-arachidonoyl glycerol (2AG) were higher in fibrotic compared to normal skin (**Figure 1E**), suggesting an upregulated endocannabinoid system in the fibrotic tissue. However, chronic MRI-1867 administration did not reduce bleomycin-induced increase in endocannabinoids (**Figure 1E**), suggesting a lack of CB_1_R engagement by MRI-1867 in the fibrotic skin of C57BL/6J mice (**Figure 1E**), due to compromised skin exposure (**Figure 1D**). The dramatic loss in skin exposure to MRI-1867 might be attributed to bleomycin-induced over-expression and over-activity of P-gp, which was observed previously in bleomycin-induced pulmonary fibrosis (27). Indeed, P-gp protein expression was increased by bleomycin in skin biopsy specimens from C57BL/6J mice (**Figures 2A, B**).

### Skin Exposure to MRI-1867 Was Recovered in Bleomycin-Induced Skin Fibrosis Using $\text{Mdr}1_{\text{a}/\text{b}}^{\left(-/-\right)}$ -Bcrp^(-/-)^ Triple Knock-Out Mice

**Figure 3:**
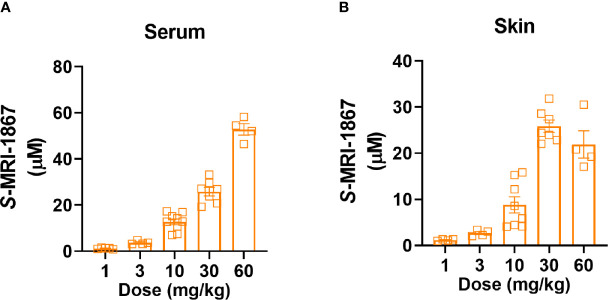
Dose-dependent systemic and skin exposure of MRI-1867 in $\text{Mdr}1_{\text{a}/\text{b}}^{\left(-/-\right)}$ -Bcrp^(-/-)^ mice. **(A)** Serum and **(B)** Skin levels of MRI-1867 at 1 hour after the last dose of 14 days oral administration at 1, 3, 10, 30, and 60 mg/kg doses in bleomycin-treated $\text{Mdr}1_{\text{a}/\text{b}}^{\left(-/-\right)}$ -Bcrp^(-/-)^ mice. Data represent means ± SEM from 4-10 mice per group.

As bleomycin causes a ~100-fold reduction in skin exposure to MRI-1867 in C57BL/6J mice, this model is unsuitable for the preclinical testing of the antifibrotic potential of MRI-1867, a known substrate of drug efflux transporters. Instead, we decided to use Mdr1_a/b_-Bcrp triple knockout mice for this purpose as a way to bypass the artifact caused by increased activity of drug efflux transporters. First, we measured the levels of MRI-1867 in the fibrotic skin of bleomycin-exposed $\text{Mdr}1_{\text{a}/\text{b}}^{\left(-/-\right)}$ -Bcrp^(-/-)^ triple KO mice after 14 days of chronic MRI-1867 treatment at 1, 3, 10, 30, 60 mg/kg doses (**Figure 3**). Levels of MRI-1867 dose-dependently increased in serum (**Figure 3A**). Importantly, skin exposure to MRI-1867 was much higher in $\text{Mdr}1_{\text{a}/\text{b}}^{\left(-/-\right)}$ -Bcrp^(-/-)^ triple KO mice compared to wild-type mice (**Figure 3B**), such that the 10 mg/kg dose of MRI-1867 achieved a concentration of 8.8 µM in the fibrotic skin (**Figure 3B**) compared to 0.12 µM in wild-type C57BL6/J mice (**Figure 1D**). Maximum skin exposure in the triple KO mice was ~26 µM following chronic treatment with the 30 mg/kg dose (**Figure 3B**). However, skin exposure was not further increased with 60 mg/kg/day dosing, which might be due to an altered ADME/PK profile with the higher dose of MRI-1867. Therefore, the 30 mg/kg/day dose was selected to explore the maximum achievable efficacy of MRI-1867 in this skin fibrosis model and to establish the PK/PD relationship.

### MRI-1867 Significantly Attenuated Dermal Thickness and Skin Fibrosis in Bleomycin-Induced Skin Fibrosis in $\text{Mdr}1_{\text{a}/\text{b}}^{\left(-/-\right)}$ Bcrp^-/-^ Knock-Out Mice

**Figure 4:**
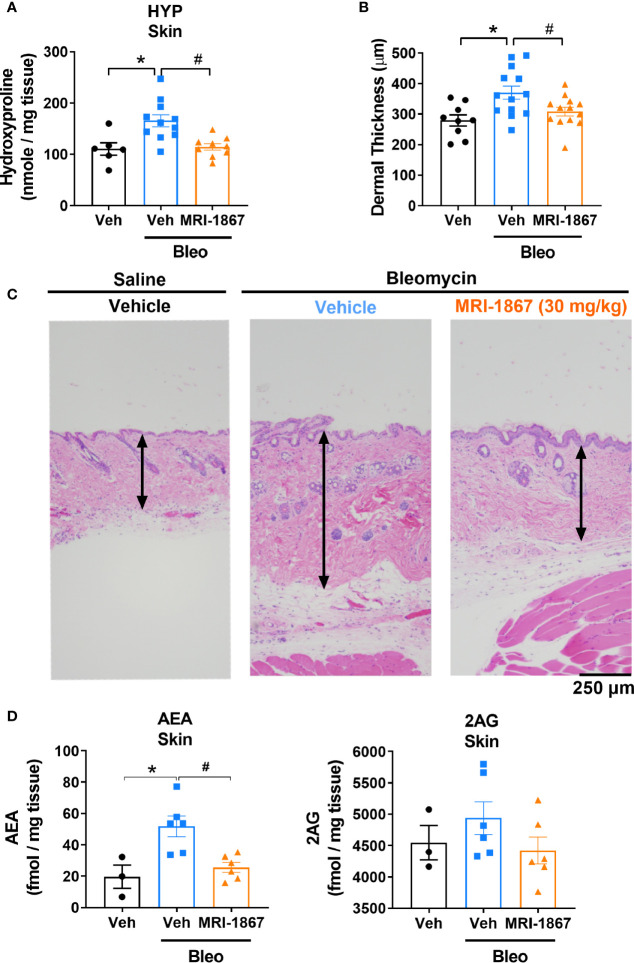
MRI-1867 (30 mg/kg) attenuates bleomycin-induced fibrosis and dermal thickness in $\text{Mdr}1_{\text{a}/\text{b}}^{\left(-/-\right)}$ -Bcrp^(-/-)^ mice. **(A)** Hydroxyproline levels in biopsied skin. **(B)** Dermal thickness analyses from skin biopsies. **(C)** Representative histological images from skin biopsies stained with H&E. **(D)** AEA and 2AG levels in biopsied skin. Six mm skin biopsies are used for the analysis. Dermal thickness assessed from left scapular injection site biopsies, Hydroxyproline and endocannabinoid measurements performed using right scapular injection site biopsies from each mouse. Data represent means ± SEM from 3-13 mice per group. Data were analyzed by 1-way ANOVA followed by Dunnett’s multiple comparisons test. Significant difference from corresponding values in control (saline and vehicle treated group) (*P < 0.05) or from values in bleomycin and vehicle-treated group (^#^P < 0.05).

Daily subcutaneous bleomycin injections for 28 days significantly increased the levels of hydroxyproline (**Figure 4A**) and dermal thickness (**Figures 4B, C**), and endocannabinoids in the fibrotic skin (**Figure 4D**). Chronic daily oral administration of MRI-1867 for the last 14 days of the 28 day bleo treatment significantly attenuated bleomycin-induced hydroxyproline (**Figure 4A**), dermal thickness (**Figures 4B, C**), and endocannabinoid levels (**Figure 4D**) in the fibrotic skin of $\text{Mdr}1_{\text{a}/\text{b}}^{\left(-/-\right)}$ -Bcrp^(-/-)^ mice, suggesting that endocannabinoid tone is reduced following treatment with MRI-1867 and that targeting CB_1_R is a putative target for fibrosis alleviation.

### MRI-1867 Has Higher Antifibrotic Efficacy Than Rimonabant in Skin Fibrosis

**Figure 5:**
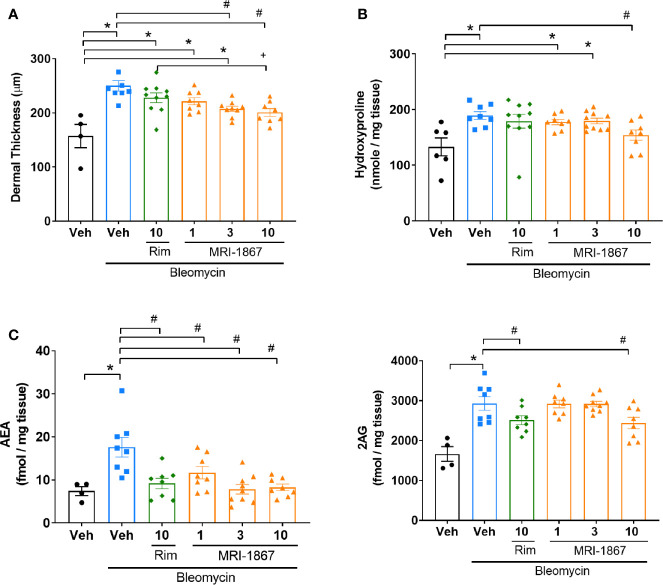
Comparison of the anti-fibrotic effects of MRI-1867 and rimonabant in $\text{Mdr}1_{\text{a}/\text{b}}^{\left(-/-\right)}$ -Bcrp^(-/-)^ mice. **(A)** Dermal thickness analysis, **(B)** Hydroxyproline levels, **(C)** AEA and 2AG levels from skin biopsies in control and bleomycin-injected Mdr1a/b(-/-) -Bcrp^(-/-)^ mice. Rimonabant (10 mg/kg) or MRI-1867 (1, 3, 10 mg/kg) were orally administered daily for the last 14 days of bleomycin injections. Data represent means ± SEM from 4-10 mice per group. Data were analyzed by 1-way ANOVA followed by Dunnett’s multiple comparisons test. Significant difference from corresponding values in control (saline and vehicle treated group) (*P < 0.05), or from values in bleomycin and vehicle-treated group (^#^P < 0.05), or from values in bleomycin and rimonabant-treated group (^+^P < 0.05).

We next compared the therapeutic efficacy of MRI-1867 and rimonabant at a 10 mg/kg dose, which was shown to provide equipotent CB_1_R antagonism (16, 23). In addition, we also tested MRI-1867 at 1 and 3 mg/kg doses to determine the minimum effective dose that provides anti-fibrotic efficacy in bleomycin-induced skin fibrosis. Ten mg/kg MRI-1867 significantly reduced bleomycin-induced dermal thickness (**Figure 5A**) and hydroxyproline content (**Figure 5B**). Furthermore, 10 mg/kg MRI-1867 significantly reduced dermal thickness compared to the rimonabant and vehicle (**Figure 5A**). However, rimonabant did not significantly reduce dermal thickness compared to the vehicle. At the dose of 1 mg/kg, MRI-1867 had no significant antifibrotic effect. While the 3 mg/kg dose significantly attenuated dermal thickness (**Figure 5A**), it did not significantly reduce hydroxyproline (**Figure 5B**). MRI-1867 dose-dependently attenuated bleomycin-induced increases in skin endocannabinoids. Both rimonabant and MRI-1867 at the 10 mg/kg dose significantly and comparably attenuated skin endocannabinoids, suggesting similar target engagement in fibrotic skin (**Figure 5C**).

## Discussion

We have discovered that using bleomycin to model skin fibrosis in C57BL/6J mice introduces an artifact related to upregulation of drug efflux transporters in skin tissue. This would confound studies aimed to test the pharmacokinetics and target engagement of drug candidates that happen to be substrates. This finding aligns with our previous work and the work of others in preclinical models of bleomycin-induced pulmonary fibrosis (27, 29), highlighting a limitation of bleomycin-induced fibrosis models. We also show that using $\text{Mdr}1_{\text{a}/\text{b}}^{\left(-/-\right)}$ -Bcrp^(-/-)^ mice for preclinical testing of such compounds would avoid this pitfall. In lung specimens from IPF patients, efflux transporters such as P-gp and BCRP were expressed at the same level as in lung samples from appropriate controls (23), which indicates that the increased P-gp expression seen in the preclinical model is not part of the pathological process in human IPF. A recent study compared the frequencies of 3 single nucleotide polymorphisms (SNPs) in the *ABCB1* gene, which encodes P-gp, and found no differences between patients with systemic sclerosis and their controls in a Polish population. Although a specific haplotype of these SNPs occurred significantly more frequently among patients than among their controls, there was no evidence presented for an association of this haplotype with altered gene or protein expression or transporter activity of ABCB1 (30).

Additionally, the present findings demonstrate that the dual-target inhibition of CB_1_R and iNOS by MRI-1867 is an effective anti-fibrotic strategy for scleroderma that warrants further study. This finding is in line with our previous studies showing that MRI-1867 can attenuate fibrosis in other organs as well, including the liver (16), kidney (28), and lungs (23, 24). This is consistent with mounting evidence that CB_1_R may be part of a core mechanism of fibrogenesis and that CB_1_R antagonism may have therapeutic potential in several fibrotic disorders, including chronic kidney (31, 32) and liver diseases (33) and cardiomyopathies (34, 35). Although MRI-1867 was more efficacious than rimonabant at equipotent doses for CB_1_R antagonism, we have not investigated the relative contribution of CB_1_R and iNOS inhibition, which may be subject to future studies. Recently, a structural analogue of MRI-1867 was identified as a β-arrestin-2 biased CB_1_R antagonist, whereas rimonabant was unbiased (36). Although we have not explored a potential signaling bias of CB_1_R activation in skin fibrosis development, the superior efficacy of MRI-1867 over rimonabant could not be attributed to functional selectivity since MRI-1867 does not display signaling bias in CB_1_R antagonism (unpublished information).

In addition to fibrosis, numerous studies have documented that an overactive endocannabinoid/CB_1_R system contributes to visceral obesity and its complications (37), including type-2 diabetes (21), and also play a role in the pathology of alcoholic liver disease (38) and viral hepatitis (39). Conversely, CB_1_R blockade has beneficial effects in preclinical models of these conditions as well as in overweight individuals with metabolic syndrome (40). However, brain-penetrant CB_1_R antagonists, such as rimonabant, cause psychiatric side effects due to the blockade of CB_1_R in the CNS, which had halted their therapeutic development. Non-brain-penetrant CB_1_R antagonists have recently been reported to retain the metabolic benefit of globally acting compounds without blocking CB_1_R in the CNS (21, 41–43). Thus, efforts to engage CB_1_Rs for mitigating fibrosis would require antagonists with limited brain exposure in order to avoid neuropsychiatric side effects, therefore peripheral dual-target CB_1_R antagonists might be an emerging therapeutic modality for metabolic and fibrotic disorders (44).

Previously it was shown that deletion of CB_1_R protected mice from bleomycin-induced skin fibrosis (25), which aligns with the current findings and supports the therapeutic potential of peripheral CB_1_R antagonism in skin fibrosis. Deletion of CB_1_R decreased the number of infiltrating T cells and macrophages in lesioned skin (25), suggesting critical roles of CB_1_R in leukocyte infiltration, inflammation, and fibroblast activation. Additionally, bone marrow transplantation from CB_1_R^-/-^ mouse into CB_1_R^+/+^ mouse protected the CB_1_R^+/+^ mice from bleomycin-induced skin fibrosis development, which implicated CB_1_R expressing myeloid cell populations in inflammation-driven skin fibrosis development (25). Additionally, CB_1_R signaling in keratinocytes also regulates T-cell dependent inflammation in skin (45). It is important to note that CB_1_R is expressed in multiple cell types in skin, and its role in skin pathologies and inflammation can be context dependent (46). This suggests that activation status and potential paracrine regulation of endocannabinoid/CB_1_R system in different cells in the local pathologic microenvironment might be critical factors for the pro-inflammatory and profibrotic activity of CB_1_Rs in skin fibrosis. Indeed, this notion is supported by our finding that a significant loss of MRI-1867 exposure in the lesioned skin resulted in loss of its anti-fibrotic efficacy despite the high level of systemic exposure. Our finding demonstrated that CB1R inhibition is required at the site of action to result in anti-fibrotic efficacy. This could suggest that topical application of CB_1_R antagonists might be a therapeutic strategy in skin fibrosis. However, systemic administration should be the preferred therapeutic approach considering its potential therapeutic benefit in multi-organ involvement in systemic sclerosis (44).

A pro-inflammatory role of CB_1_R resulting in macrophage activation was established in the pancreas during diabetes and in the lung during pulmonary fibrosis (21, 23). Furthermore, interferon regulatory factor 5 (IRF5) was found to be an essential down-stream mediator of CB_1_R signaling in macrophages in diabetes (47) and transplantation of CB_1_R^-/-^ bone marrow to pre-diabetic ZDF rats prevented β-cell loss and diabetic complications, supporting the pathogenic role CB_1_R-mediated IRF5 signaling. IRF5 is a master regulator of pro-inflammatory macrophages. Furthermore, IRF5 polymorphism increases the risk of systemic sclerosis whereas reduced expression of IRF5 increases survival (48, 49). Indeed, deletion of IRF5 protected mice from development of bleomycin-induced skin and pulmonary fibrosis (50), which makes IRF5 a potential therapeutic target in systemic sclerosis and scleroderma. Deletion of CB_1_R also attenuated bleomycin-induced increase in IRF5 in lungs and protected from pulmonary fibrosis (23). Thus, the intriguing possibility that CB_1_R-mediated IRF5 signaling may contribute to skin and pulmonary fibrosis development in systemic sclerosis and scleroderma, needs to be explored in future studies.

Interestingly, targeting CB_1_R may also be promising for symptom management. One of the most common symptoms of systemic sclerosis patients that affects quality of life is gastrointestinal dysmotility, which results in constipation (51). Previously, we found that MRI-1867 increases upper gastrointestinal motility in mice *via* peripheral CB_1_R inverse agonism (16, 52), which might compensate for constipation. In summary, the present findings introduce a polypharmacology approach to the treatment of skin fibrosis whereby simultaneous engagement of two therapeutic targets by a single molecule is harnessed for improved therapeutic efficacy. Clinical studies in scleroderma patients are warranted once MRI-1867 or related compounds become available for human studies.

## Author Contributions

CZ and JP performed *in vivo* experiments, histology, immunohistochemistry, image acquisition and analysis, and participated in manuscript preparation. JA contributed immunohistochemistry. MRI synthesized and chemically analyzed MRI-1867 and contributed manuscript preparation and study concept. RC designed the study, planned experiments, performed mass spectrometry experiments, analyzed and interpreted data, and drafted the manuscript. GK reviewed data and finalized the manuscript. All authors contributed to the article and approved the submitted version.

## Funding

This work was supported by the Intramural Research Programs of the NIAAA and by a Collaborative Research and Development Agreement (CRADA) with SCOPUS BIOPHARMA INC., NY, USA.

## Conflict of Interest

This study received funding from SCOPUS BIOPHARMA INC., NY, USA. The funder was not involved in the study design, collection, analysis, interpretation of data, the writing of this article or the decision to submit it for publication. RC, GK, and MRI are listed as coinventors on a US patent covering MRI-1867 and related compounds (patent no. US 9,765,031 B2).

The remaining authors declare that the research was conducted in the absence of any commercial or financial relationships that could be construed as a potential conflict of interest.

## Publisher’s Note

All claims expressed in this article are solely those of the authors and do not necessarily represent those of their affiliated organizations, or those of the publisher, the editors and the reviewers. Any product that may be evaluated in this article, or claim that may be made by its manufacturer, is not guaranteed or endorsed by the publisher.
